# Supplementary material for: Direct in-situ imaging of electrochemical corrosion of Pd-Pt core-shell electrocatalysts
Source: Nat Commun. 2024 Jun 14;15:5084. doi: 10.1038/s41467-024-49434-3 (PMC11178921; doi:10.1038/s41467-024-49434-3)
Supplement: Supplementary file 1 — Supplementary Information [file 41467_2024_49434_MOESM1_ESM.pdf]

Supplementary Information

Direct In-Situ Imaging of Electrochemical Corrosion of Pd-Pt Core-Shell Electrocatalysts

Fenglei Shi,[1#] Peter Tieu,[2#] Hao Hu,[1#] Jiaheng Peng,[1] Wencong Zhang,[1] Fan Li,[1] Peng Tao,[1] Chengyi Song,[1] Wen Shang,[1] Tao Deng,[1] Wenpei Gao,[1,3,*] Xiaoqing Pan,[4,5,*] and Jianbo Wu[1,3,6,*]

[1] Center of Hydrogen Science & State Key Laboratory of Metal Matrix Composites, School of Materials Science and Engineering, Shanghai Jiao Tong University, 800 Dongchuan Rd, Shanghai, 200240, People's Republic of China
[2] Department of Chemistry, University of California, Irvine, Irvine, CA, 92697, USA
[3] Future Material Innovation Center, Zhangjiang Institute for Advanced Study, Shanghai Jiao Tong University, Shanghai 200240, People's Republic of China.
[4] Department of Materials Science and Engineering, University of California, Irvine, Irvine, CA, 92697, USA
[5] Department of Physics and Astronomy, University of California, Irvine, Irvine, CA, 92697, USA
[6] Materials Genome Initiative Center, Shanghai Jiao Tong University, Shanghai, People's Republic of China

Correspondence and request for materials should be addressed to J.W., X. Pan, and W. Gao.

*Email: jianbowu@sjtu.edu.cn; xiaoqinp@uci.edu; gaowenpei@sjtu.edu.cn

Contents

Figures

Supplementary Fig. 1 TEM images of the nanoparticles after different steps during the synthesis.
Supplementary Fig. 2 XRD patterns of Pd@Pt octahedral nanoparticles.
Supplementary Fig. 3 Time-sequential in-situ TEM images of Pd@Pt octahedral nanoparticles under -0.5 V to 0.5 V (vs. Pt) CV conditions.
Supplementary Fig. 4 HAADF-STEM images and element analysis of nanoparticles after in-situ electrochemical CV (-0.5 V to 0.5 V vs. Pt) treatment.
Supplementary Fig. 5 Time-sequential in-situ TEM images of Pd@Pt octahedral nanoparticles under -0.2 V to 0.2 V (vs. Pt) CV conditions.
Supplementary Fig. 6 HAADF-STEM and EDS characterizations of Pd@Pt core-shell nanoparticles after in-situ electrochemical CV (-0.2 V to 0.2 V vs. Pt) treatment.
Supplementary Fig. 7 Time-sequential in-situ TEM images of a single Pd@Pt octahedron under -0.9 V to -0.2 V (vs. Pt) CV conditions.
Supplementary Fig. 8 Surface steps formation during the ex-situ electrochemical ADT.
Supplementary Fig. 9 HAADF-STEM image of the nanoparticle after 30000 ADT cycles.
Supplementary Fig. 10 HAADF-STEM images of nanoparticles after 50000 ADT cycles exhibited different degrees of nanoframe morphology.
Supplementary Fig. 11 HAADF-STEM image and EDS mapping of carbon support after 60000 ADT cycles.
Supplementary Fig. 12 Time-sequential in-situ TEM images of Pd@Pt octahedral nanoparticles without electrochemical conditions.
Supplementary Fig. 13 Calibration of Pt wire reference electrode using RHE.
Supplementary Fig. 14 Experimental set-up of the in-situ TEM.

Table

Supplementary Table 1 ICP-AES and TGA results.

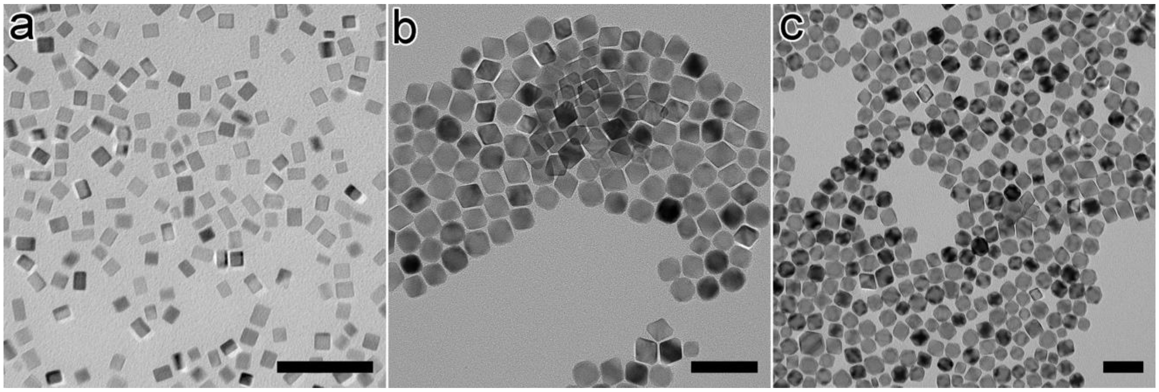

Supplementary Fig. 1 TEM images of the nanoparticles after different steps during the synthesis. a, Pd cubic seeds (step one). b, Pd octahedral seeds (step two). c, Pd@Pt octahedral nanoparticles (step four). The scale bars are 50 nm.

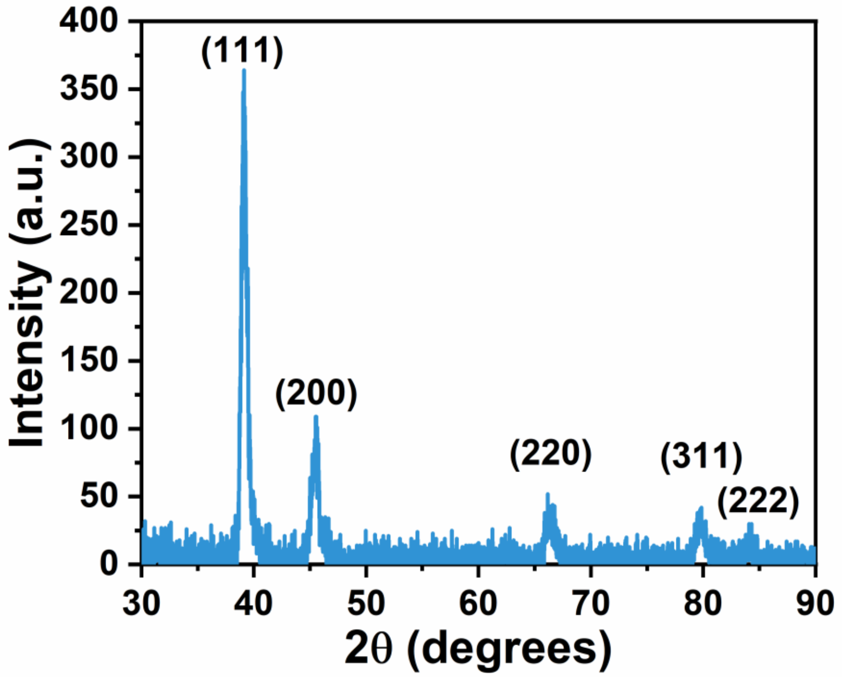

Supplementary Fig. 2 XRD patterns of Pd@Pt octahedral nanoparticles.

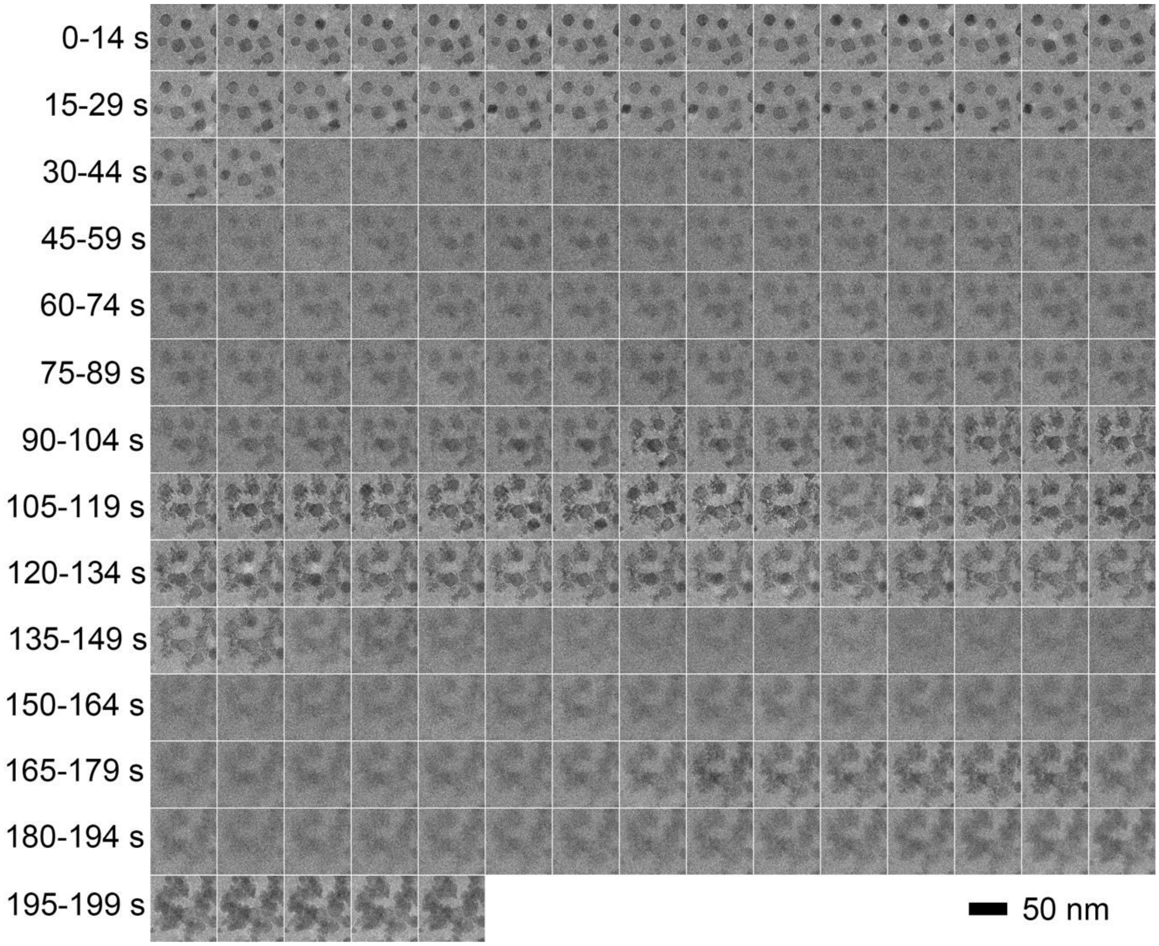

Supplementary Fig. 3 Time-sequential in-situ TEM images of Pd@Pt octahedral nanoparticles under -0.5 V to 0.5 V (vs. Pt) CV conditions. Some TEM images were ambiguous due to the turbulence in the liquid cell during the electrochemical process.

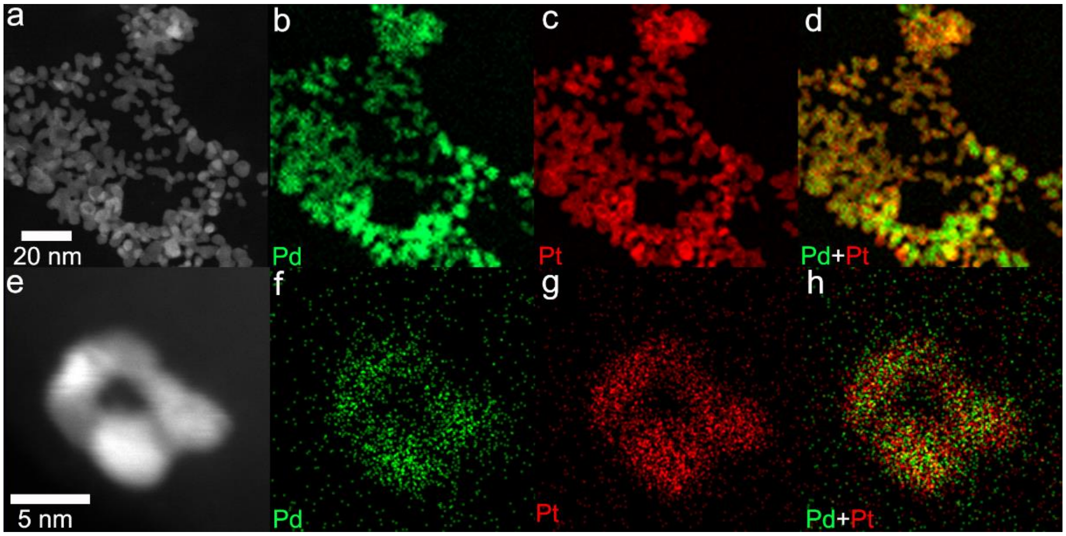

Supplementary Fig. 4 HAADF-STEM images and element analysis of nanoparticles after in-situ electrochemical CV (-0.5 V to 0.5 V vs. Pt) treatment. a, e, Atomic-resolution STEM images of a Pd@Pt octahedron after in-situ electrochemical CV (-0.5 V to 0.5 V vs. Pt) treatment. b-d, f-h, EDS mapping of a Pd@Pt octahedron after in-situ electrochemical CV (-0.5 V to 0.5 V vs. Pt) treatment. The green and red colors correspond to Pd and Pt elements, respectively.

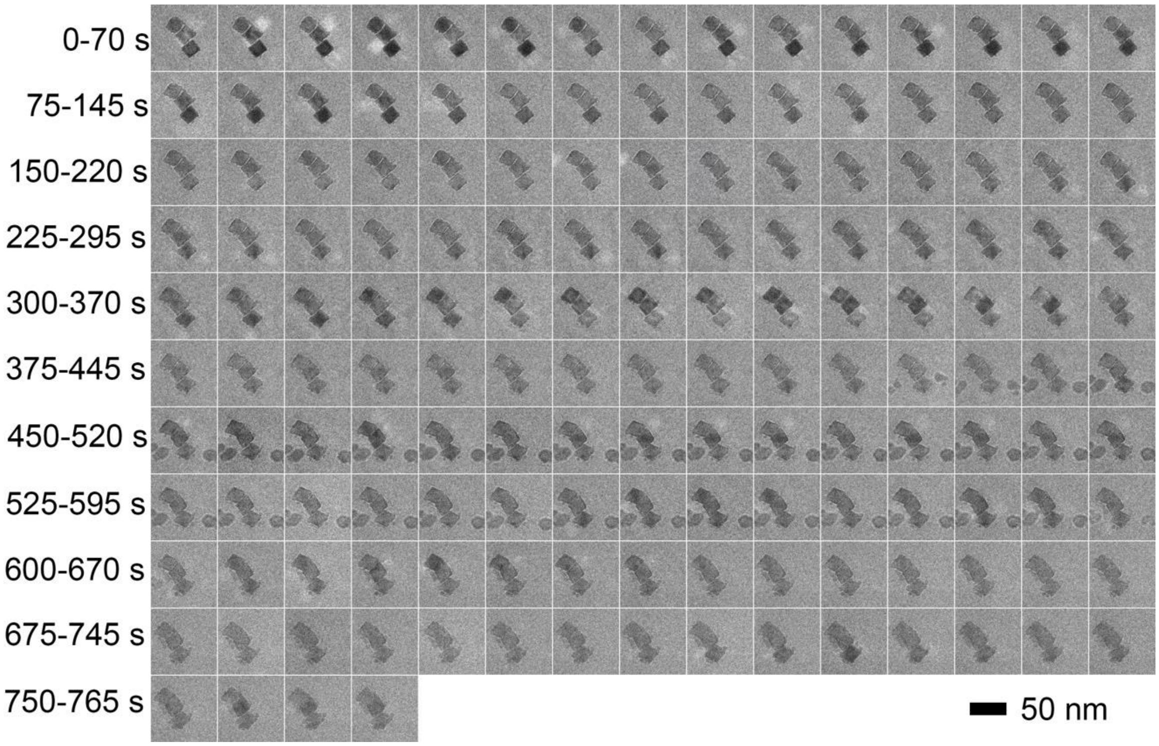

Supplementary Fig. 5 Time-sequential in-situ TEM images of Pd@Pt octahedral nanoparticles under -0.2 V to 0.2 V (vs. Pt) CV conditions. There were some small nanoparticles floating into the field of vision (435 s to 595 s). These small nanoparticles and the octahedra were not at the same height and were carried by the turbulence in the liquid cell.

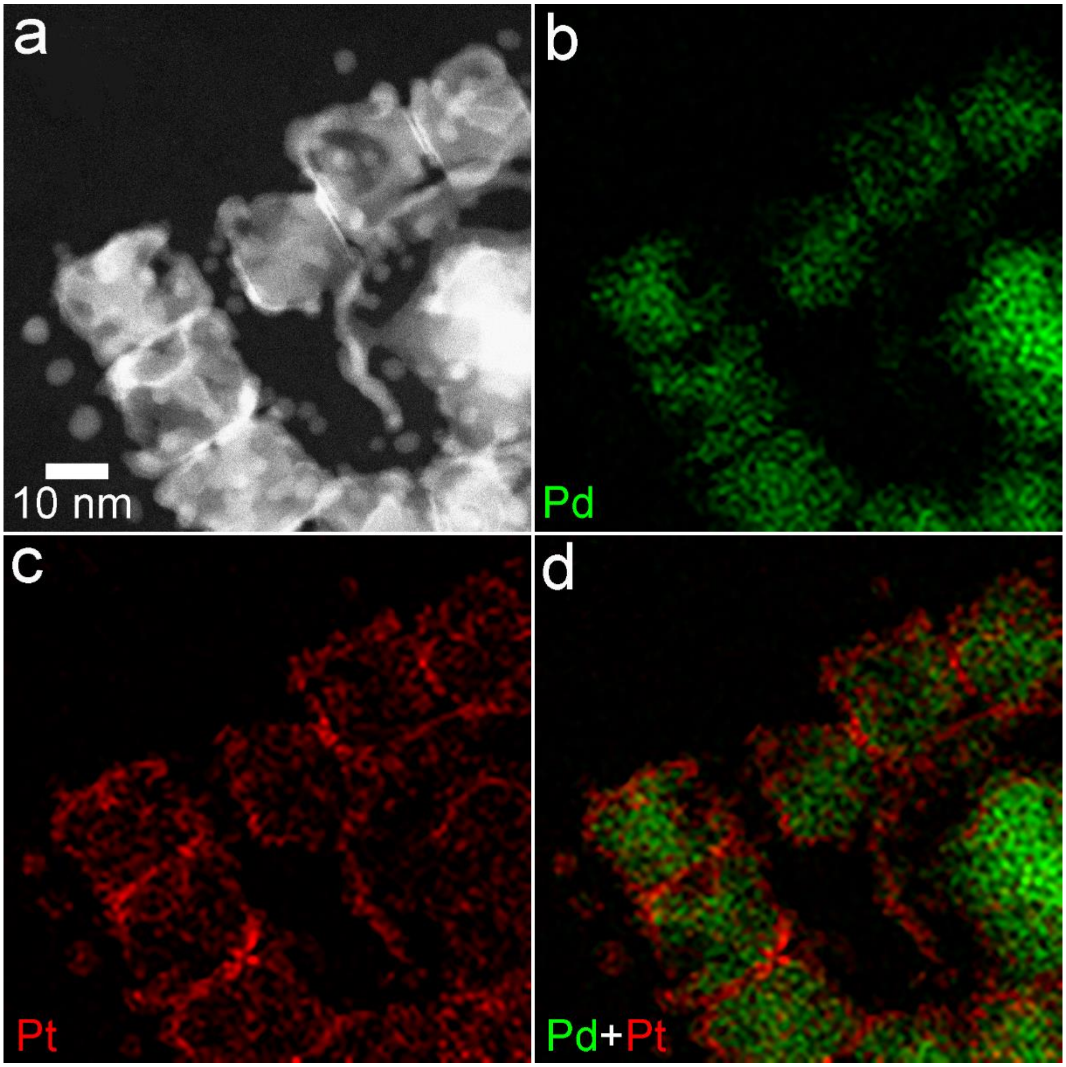

Supplementary Fig. 6 HAADF-STEM and EDS characterizations of Pd@Pt core-shell nanoparticles after in-situ electrochemical CV (-0.2 V to 0.2 V vs. Pt) treatment. a, HAADF-STEM image of Pt surface islands reduced on the nanoparticles. b-d, EDS mapping of nanoparticles after in-situ electrochemical CV (-0.2 V to 0.2 V vs. Pt) treatment. The green and red colors correspond to Pd and Pt elements, respectively.

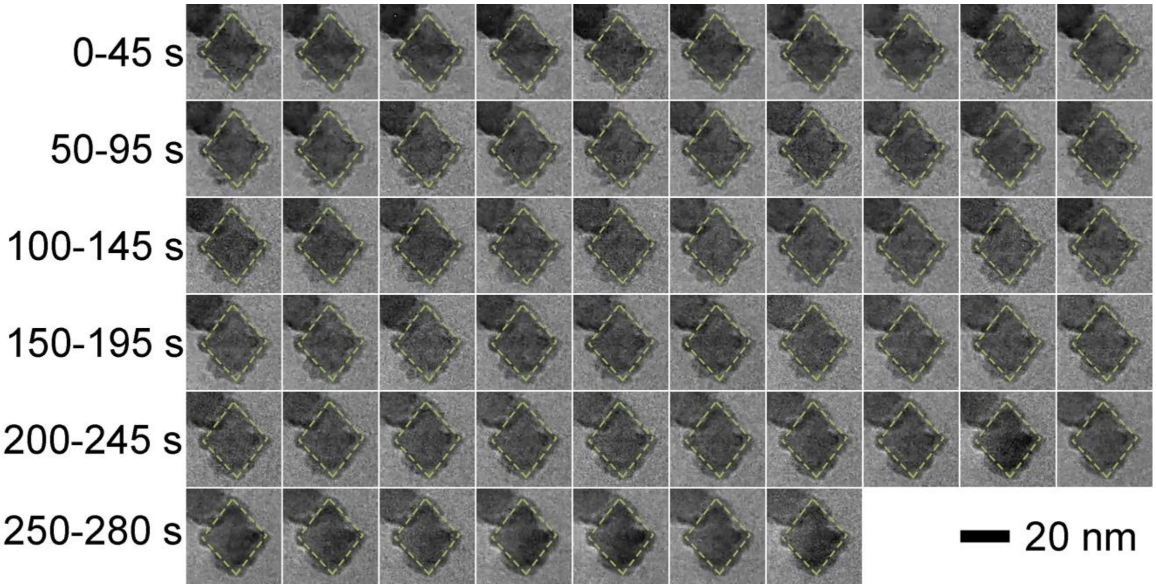

Supplementary Fig. 7 Time-sequential in-situ TEM images of a single Pd@Pt octahedron under -0.9 V to -0.2 V (vs. Pt) CV conditions. Choosing the appropriate CV potential range is more favorable for us to observe and investigate the corrosion process.

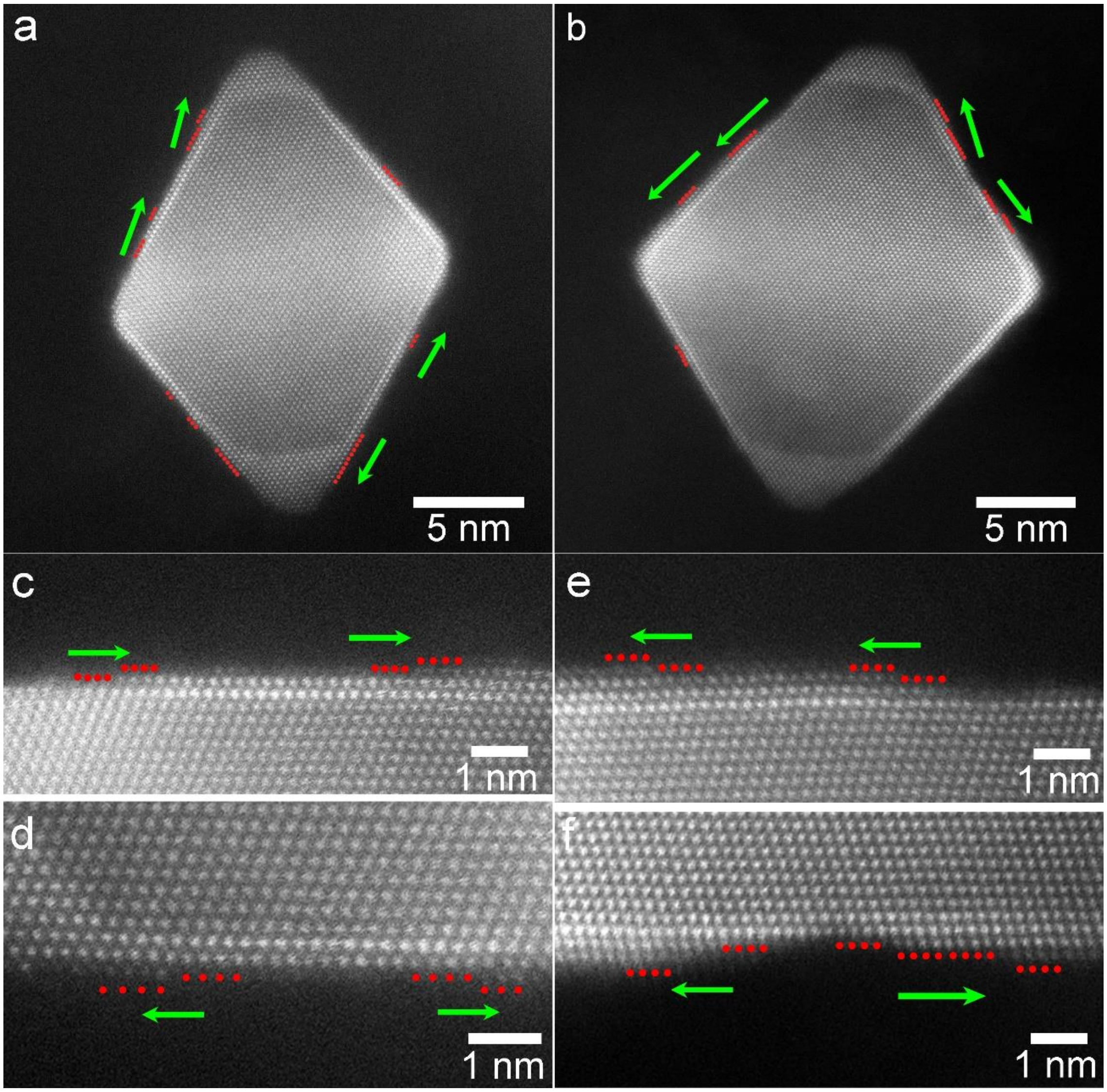

Supplementary Fig. 8 Surface steps formation during the ex-situ electrochemical ADT. a-b, HAADF-STEM images of the nanoparticles after 10000 ADT cycles. c-d, Enlarged HAADF-STEM images of the nanoparticles in Supplementary Fig. 8a. e-f, Enlarged HAADF-STEM images of the nanoparticles in Supplementary Fig. 8b. The red dots in all TEM images represented the formed surface steps. Green arrows showed the directions of decreasing Pt layers.

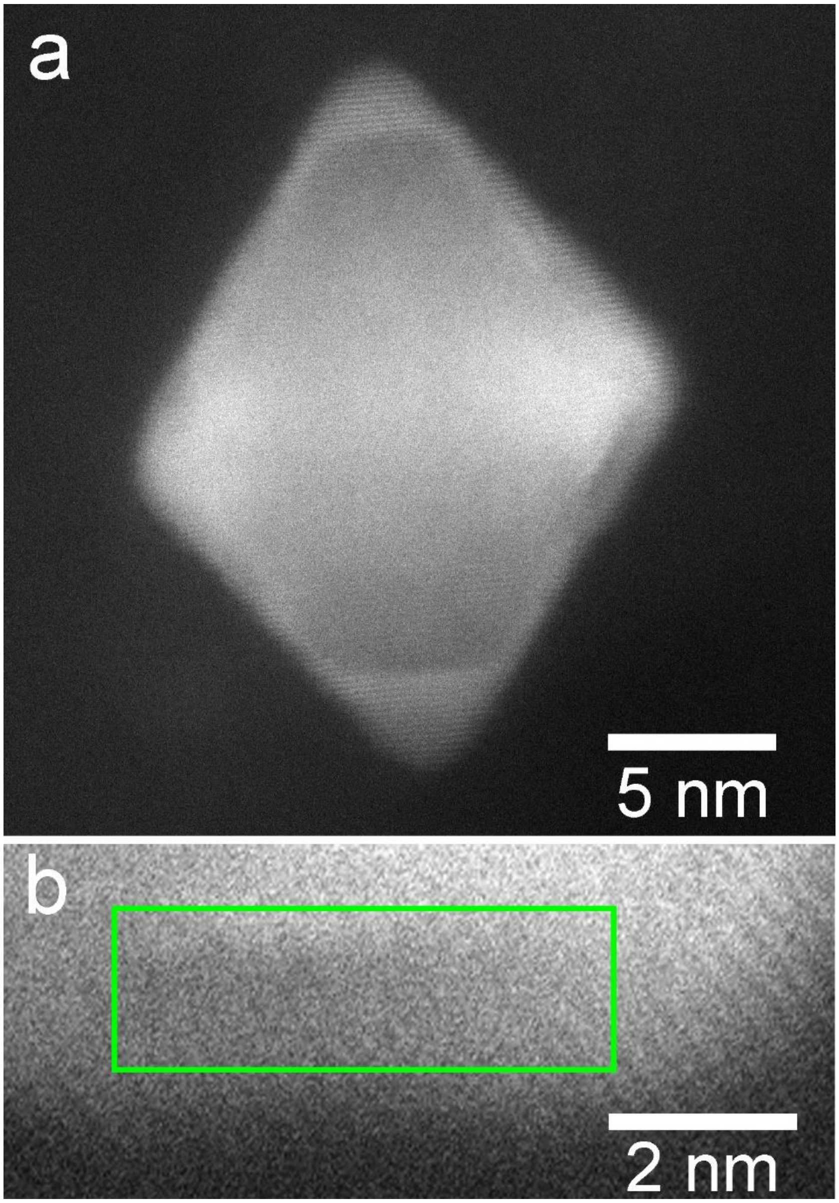

Supplementary Fig. 9 HAADF-STEM image of the nanoparticle after 30000 ADT cycles. a, HAADF-STEM image of the nanoparticle in Fig. 4i in a different orientation, showing the etching of inner Pd atoms (green arrow, corresponding to Fig. 4l). b, Enlarged HAADF-STEM image of the nanoparticle in Fig. S9a, showing the etching region of the inner Pd (green rectangle).

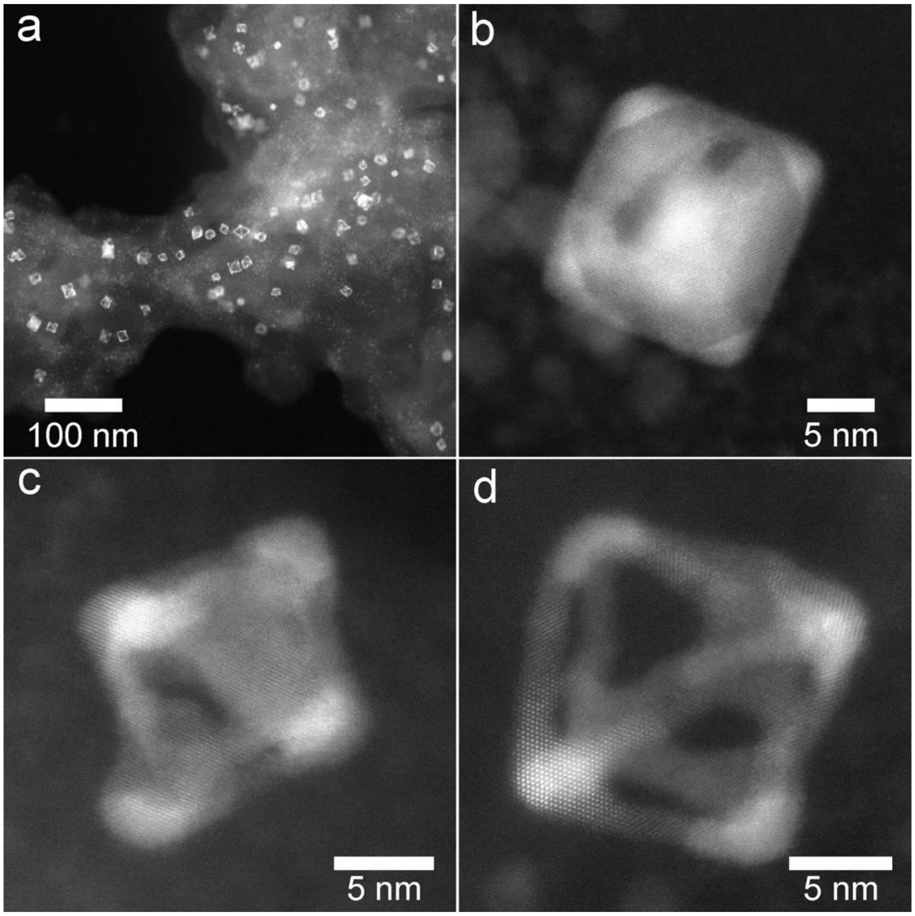

Supplementary Fig. 10 HAADF-STEM images of nanoparticles after 50000 ADT cycles exhibited different degrees of nanoframe morphology. a, Wide view of nanoparticles after 50000 ADT cycles. b-d, Increasing degree of nanoframe formation.

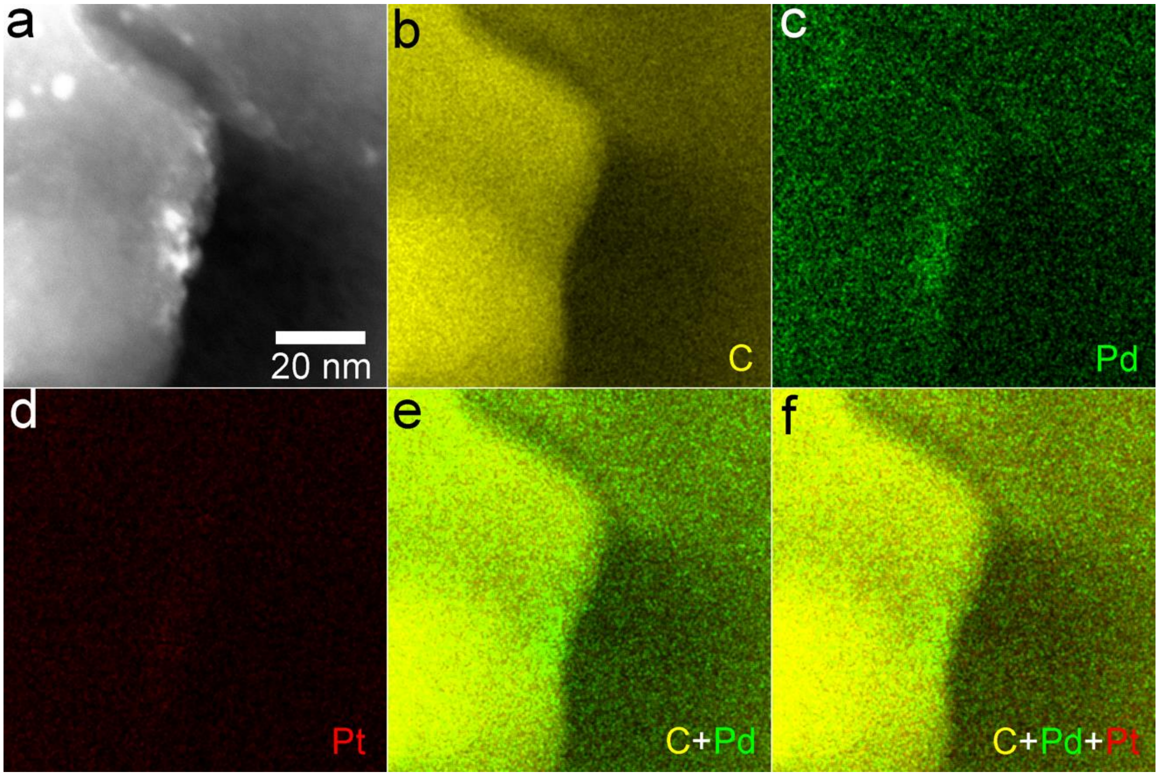

Supplementary Fig. 11 HAADF-STEM image and EDS mapping of carbon support after 50000 ADT cycles. a, HAADF-STEM image of Pd element reduced on carbon support. b-f, EDS mapping of carbon support after 50000 ADT cycles. The green, red and yellow colors correspond to Pd, Pt, and carbon elements, respectively.

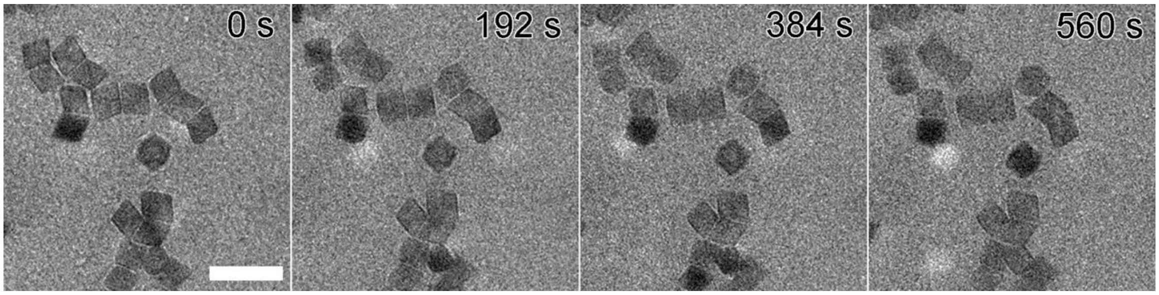

Supplementary Fig. 12 Time-sequential in-situ TEM images of Pd@Pt octahedral nanoparticles without electrochemical conditions showed that the electron beam didn't damage the materials. The scale bar was 50 nm. The dose rate we used in all the in-situ experiments was 50 ~ 60 e/Å^2·s.

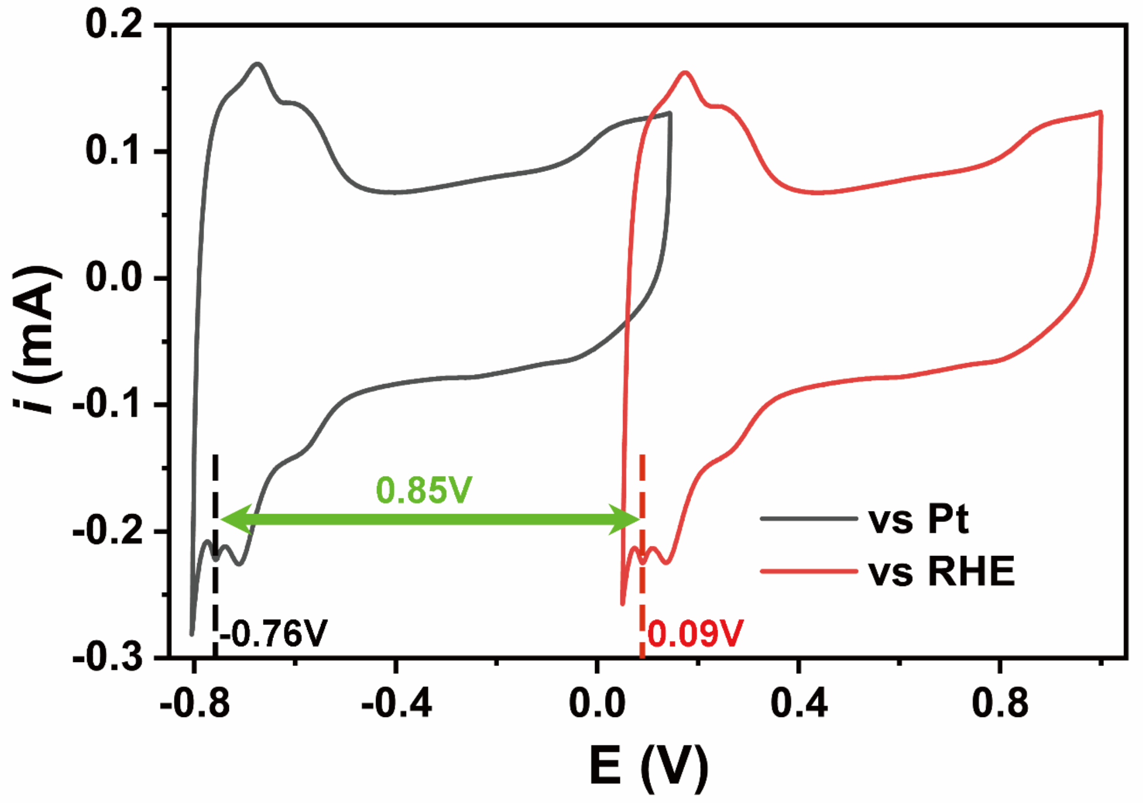

Supplementary Fig. 13 Calibration of Pt wire reference electrode using RHE.

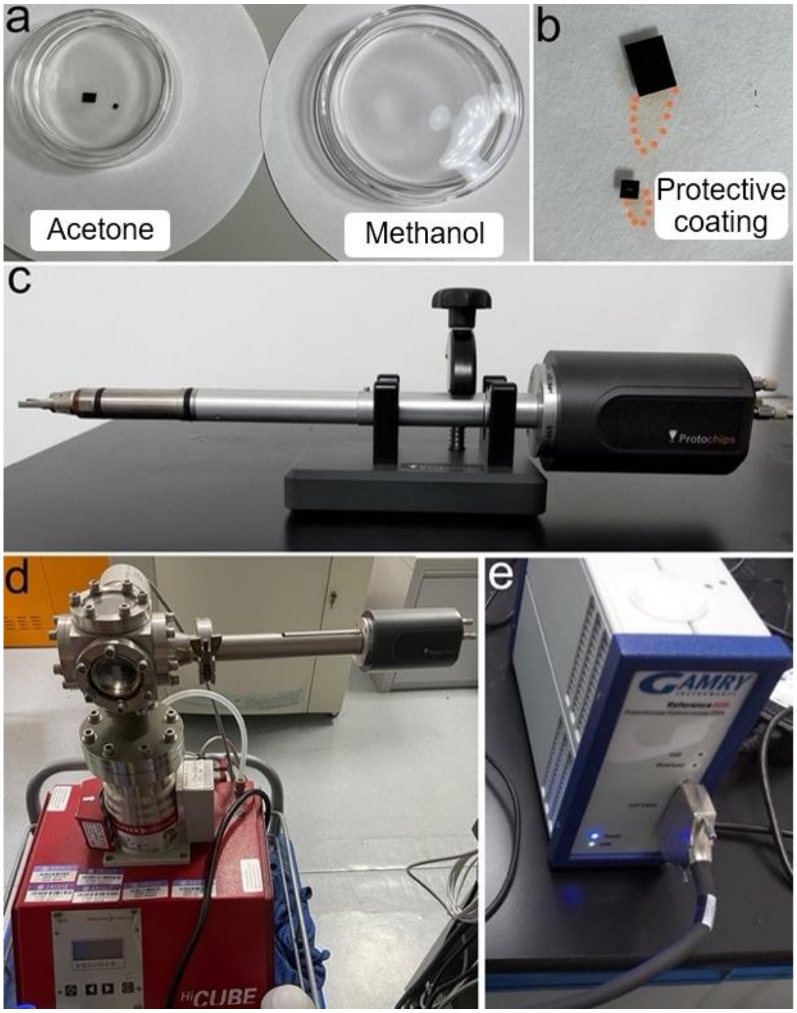

Supplementary Fig. 14 Experimental set-up of the in-situ TEM. a-b, Removal of the protective E-chip coating. c, Poseidon Select TEM holder. d, Leak check in a vacuum pump. e, Electrochemical workstation (Gamry, Reference 600).

Supplementary Table 1 ICP-AES and TGA results.

Mass ratio	Pd : Pt	Pd@Pt : C
ICP-AES	69.3 : 30.7	
TGA		0.23 : 0.77
