# Supplementary material for: Direct in-situ imaging of electrochemical corrosion of Pd-Pt core-shell electrocatalysts
Source: Nat Commun. 2024 Jun 14;15:5084. doi: 10.1038/s41467-024-49434-3 (PMC11178921; doi:10.1038/s41467-024-49434-3)
Supplement: Supplementary file 3 — Description of Additional Supplementary Files [file 41467_2024_49434_MOESM3_ESM.pdf]

Description of Additional Supplementary Files

Supplementary Movie 1: Corrosion process of Pd@Pt octahedral nanoparticles under -0.5 V to 0.5 V CV conditions.

Supplementary Movie 2: Corrosion process of Pd@Pt octahedral nanoparticles under -0.2 V to 0.2 V CV conditions.

Supplementary Movie 3: Corrosion process of a single Pd@Pt octahedron under -0.9 V to -0.2 V CV conditions.
